# Ethnicity and health inequalities: an empirical study based on the 2010 China survey of social change (CSSC) in Western China

**DOI:** 10.1186/s12889-020-08579-8

**Published:** 2020-05-07

**Authors:** Y. J. Wang, X. P. Chen, W. J. Chen, Z. L. Zhang, Y. P. Zhou, Z. Jia

**Affiliations:** 1 College of Earth and Environmental Sciences, Lanzhou University, Lanzhou, 730000 China; 2 Research Center for Circular Economy in Western China, Lanzhou University, Lanzhou, 730000 China; 3 Philosophy and Sociology School of Lanzhou University, Lanzhou, 730000 China; 4 Key Laboratory of Western China’s Environmental Systems (Ministry of Education), Lanzhou University, Lanzhou, 730000 China

**Keywords:** Ethnic disparity, Self-rated health, Health inequality, Western China

## Abstract

**Background:**

In China, ethnic minorities often live in frontier areas and have a relatively small population size, and tremendous social transitions have enlarged the gap between eastern and western China, with western China being home to 44 ethnic minority groups. These three disadvantages have health impacts. Examining ethnicity and health inequality in the context of western China is therefore essential.

**Methods:**

This paper is based on data from the 2010 China Survey of Social Change (CSSC2010), which was conducted in 12 provinces, autonomous regions and province-level municipalities in western China and had a sample size of 10,819. We examined self-rated health and disparities in self-rated health between ethnic minorities and Han Chinese in the context of western China. Self-rated health was coded as poor or good, and ethnicity was coded as ethnic minority or Han Chinese. Ethnic differences in self-rated health was examined by using binary logistic regression. Associations among sociodemographic variables, SES variable, health behaviour variable, health problem variables and self-rated health were also explored.

**Results:**

Fourteen percent of respondents reported their health to be poor. A total of 15.75% of ethnic minorities and 13.43% of Han Chinese respondents reported their health to be poor, indicating a difference in self-rated health between ethnic minorities and Han Chinese. Age, gender, marital status, education, alcohol, and health problems were the main factors that affected differences in self-rated health.

**Conclusion:**

In western China, there were obvious ethnic disparities in self-rated health. Elderly ethnic minorities, non-partnered ethnic minorities, ethnic minorities with an educational level lower than middle school, and ethnic minorities with chronic disease had higher odds of poor self-rated health.

## Background

Health inequality issues have been well documented in developed countries for decades but have been relatively less reported in developing countries. There is an urgent need for studies of health inequality in developing countries, especially China, due to the goals of social stabilization and the 2030 Agenda for Sustainable Development. Health inequalities exist across different countries and regions and even among individuals. Undeniably, the health status of a population improves along with economic development and bio-tech advances; however, low-income economies still lag behind high-income economies in terms of health issues, and health inequalities even exist within high-income economies [1, 2]. In America, researchers tend to focus on race-related disparities in health, whereas European researchers prefer to elucidate the socioeconomic status and class contributions to health inequalities. In China, studies are less focused on race-related disparities in health issues.

### Racial disparities in health inequality

To date, race has received extensive attention in the health geography, sociology and public health literature in relation to health inequalities, and racial disparities have been shown to exist across health outcomes [3–11]. However, the dispute about what race is has posed a fundamental challenge to these study findings [12–14]. W.E.B. Du Bois challenged the traditional theory of the biological interpretation of health inequalities and suggested that ethnic disparities in health inequality are based on social factors rather than natural physiological differences [15]. Link and Phelan found that social conditions are the fundamental cause of differences in disease rates; people with higher SES have lower disease risk factors than people with lower SES [16]. In China, a sizeable body of literature has documented health inequalities based on socioeconomic status and the urban/rural divide. These study findings demonstrated that SES inequalities existed across health outcomes and that rural residents had less access to health care resources than their urban counterparts [17–24].

Although the interpretation of health inequalities based on race has generated extensive disputes, it cannot be ignored that racial inequalities in health have persisted over time worldwide despite progress in economic development and bio-tech advances. In China, the overall health status of ethnic minorities has significantly improved since the founding of the People’s Republic of China (PRC) in 1949; however, continued lags in the health, mortality and life expectancy of ethnic minorities call for urgent attention. Qian explored the mortality rates of 38 ethnic minorities based on the third national census data in 1982 and found that the total mortality rate of the 38 ethnic minorities was 9.47‰, which was 3.11‰ higher than the national average level; among the 38 ethnic minorities, only the Manchu mortality rate was lower than the national average rate [25]. Chen found that the ethnic minority infant mortality rate was 50.95‰ in 1990, which decreased to 46.06‰ in 2000; notably, the De’ang, Hani, Jino, Lahu, Nu, and Va ethnic minority infant mortality rates were still more than 100‰ in 2000 [26].

### Economic factors associated with health inequality

Health inequality among poor and disadvantaged groups is a long-standing but still current issue that has drawn extensive attention for decades. Such individuals generally experience worse health status and die earlier than wealthier or more privileged groups [27]. Health inequality occurs across different countries, regions, nationalities/ethnicities, and even individuals [28, 29]. Research on European countries and the United States has indicated that ethnic minorities generally report poorer self-rated health than ethnic majorities [30–33].

The most prominent explanation for ethnic health inequalities is the interaction between ethnicity and socioeconomic status/spatial inequalities among ethnic groups [32, 34–36] rather than genetic differences [31]. Poverty, low income, lower-status employment, and employment grade are the factors that have been most explored in relation to health inequality [37, 38]. In particular, SES is an important indicator of health [39–42]. People with high SES often have access to a wide range of resources, such as knowledge, money, reputation, power, and social connections. All these factors have a positive impact on their health [27]. Those with low SES might have worse health status [23]. In addition, adults with low SES have higher chances of having psychological diseases, such as depression. The same trend has also been observed among children. Children from poor families have higher chances of depression than those from wealthy families [43]. The more pronounced individuals’ income inequality is in their childhood, the lower their self-rated health as adults [44].

In terms of the effect of income inequality on health outcomes, a large body of literature has examined the negative effects of income inequality on population health since the 1970s [45–49]. Early researchers explored the association between health outcomes and income inequality using the Gini coefficient. However, different conceptualizations and measurements of income inequality, such as those based on absolute income, relative income, deprivation and social capital, may reflect different relations between health outcomes and income inequality.

### Social factors associated with health inequality

Further, other explanations relate social network factors to health outcomes. Studies of the effects of social networks on health also emerged in the 1970s through the work of innovators such as Cassel, Cobb, and Berkman, who theorized or empirically demonstrated that social networks could affect mortality [50–54]. Namely, there are dyadic effects (e.g., two spouses) [55, 56], supra-dyadic effects (e.g., infectious disease or a fad) [57], and neighbourhood effects on health [58]. Schulz’s research on the Detroit metropolitan area indicated that the spatial factor of residential isolation affected access to and utilization of political, economic, and social resources by African Americans, thereby affecting their political participation, employment, education, public community infrastructure and safety and ultimately affecting their health status [59] .

### Health inequality in China

In China, rapid globalization, industrialization, and urbanization and the ageing of the population seen during this transition have had an enormous impact on the health and spatial distribution of health care resources across different regions and population groups, resulting in significant health inequality [60]. Residents in western China tend to have worse health than their counterparts in southeastern coastal regions [61]. Although the overall heath level of the entire country has improved, there is still a gap between wealthier and poorer provinces. For example, the life expectancies of residents of Beijing and Shanghai from 1981 to 2000 increased from 71.9 to 76.1 and from 72.9 to 78.1 years, respectively, whereas the life expectancy of residents of Gansu Province increased only by 1.4 years from 66.1 to 67.5 years [62]. Studies on the population health levels in eastern, central and western China have indicated profound differences among different regions. Economic growth has exacerbated rather than mitigated these differences [63, 64]. In terms of the distribution and utilization of health care resources, the gap between urban and rural residents is also significant [18]. The unequal distribution of health care resources and low accessibility of health services worsens the health status of poor and disadvantaged groups. Resource allocation disparities between urban and rural areas embodied with China’s urban-rural binary social structure have left rural residents with less access to health care resources than their urban counterparts [19, 65].

In addition, the ethnic and cultural diversity within China makes health inequalities more complicated and more intertwined with the wax and wane of the economy. Therefore, health inequalities among different ethnic groups are an issue that cannot be neglected. Ethnic minorities in China are mostly located in remote and poor regions. Ethnic minorities mainly live in remote and mountainous geographic locations, which makes their lives difficult. Poston and Shu’s demographic study of 15 ethnic groups in China based on the dissimilarity index showed that the dissimilarity indexes for the educational levels of Manchu and Mongolians compared with those of Han Chinese were the smallest (i.e., the most similar to Han Chinese), whereas the dissimilarity indexes of Tibetans and Hani were the largest [66]. Based on the third and fourth national census data, Zhang studied the child health conditions of 42 ethnic minorities in China, indicating that the child mortality rate of ethnic minorities was twice the national child mortality rate [67].

Based on evidence from the literature, we found that the existing empirical studies on the health status of ethnic minorities have mostly been conducted in the fields of epidemiology, public health, psychology, sociology and human geography [68–71]. However, studies on the ethnic disparities of health inequality in western China are rare. There are two main reasons for this lack of research. First, there is a lack of data, and large-scale surveys on the health status of ethnic minorities in western China are scarce. Second, according to the sixth national census of China, ethnic minorities account for 8.4% of the total population of the People’s Republic of China. Restricted by their sample sizes, most sampling surveys have not been able to provide enough valid samples, resulting in a very low proportion of ethnic minorities in the final effective samples; therefore, they could not meet the requirements for statistical inference.

The 2010 China Survey of Social Change (CSSC2010) does meet the above conditions mentioned in the last paragraph. Therefore, this study used data from CSSC2010 to examine ethnic health inequality in western China. There are 12 provinces/autonomous regions/province-level municipalities with a total area of 6.86 million square kilometres in western China, and 44 ethnic minority groups inhabit this area. Nearly 40 years of profound social economic transitions have made the health issues of ethnic minorities much more complicated and have led to growing economic inequality between eastern and western China. The primary aim of this study was to examine ethnic differences in self-rated health, so the following hypothesis was proposed: H1, ethnic minorities’ self-rated health is worse than that of Han Chinese. The secondary aim was to examine associations among sociodemographic variables, SES variables, health behaviour variables, health problem variables and self-rated health. Based on the literature described above, this study proposed the following hypotheses, as shown in Table 1.

**Table 1:** List of Hypotheses

Primary hypothesis	H1	Ethnic minorities’ self-rated health is worse than that of Han Chinese
Secondary hypotheses	H2	Older people are more likely than younger people to report poor self-rated health
	H3	Women are more likely than men to report poor self-rated health
	H4	Non-partnered people are more likely than partnered people to report poor self-rated health
	H5	People with higher education levels are more likely than those with lower education levels to report good self-rated health
	H6	People with high subjective social status are more likely than those with low subjective social status to report good self-rated health
	H7	Non-smokers are more likely than smokers to report good self-rated health
	H8	Non-drinkers are more likely than drinkers to report good self-rated health
	H9	People with low physical exercise frequency are more likely than those with high physical exercise frequency to report poor self-rated health
	H10	People with chronic disease are more likely than those without chronic disease to report poor self-rated health

## Methods

### Data

We drew on data from the 2010 China Survey of Social Change (CSSC2010), a project hosted by Xi’AN Jiaotong University in Shaanxi Province and collaboratively conducted by Lanzhou University in Gansu Province, Guangxi University for Nationalities in Guangxi Zhuang Autonomous Region, Qinghai Normal University in Qinghai Province, Southwestern University of Finance and Economics in Sichuan Province, Xinjiang University in Xinjiang Uygur Autonomous Region, Lhasa Normal College in Tibet Autonomous Region, Guizhou Minzu University in Guizhou Province, Chongqing Normal University in Chongqing Municipality, North Minzu University in Yunnan Province, Ningxia University in Ningxia Hui Autonomous Region and Inner Mongolia University in Inner Mongolia Autonomous Region. This panel survey covered 12 provinces, autonomous regions and province-level municipalities in western China.

The CSSC2010 was designed to collect data on economic development and social transformation in western China, including population and health, family and intergenerational relations, social networks, social values, religion, environmental problems and others [72]. The CSSC2010 was a cross-sectional regional representative survey that was conducted in 2010 by using probability proportional to size (PPS) sampling, with a total sample of 10,946 households living in western China (12 provinces, autonomous regions or province-level municipalities). Oral consent was obtained from respondents aged above 18 within the households to complete a self-reported questionnaire. Missing data are inevitable in practice. According to Rubin’s theory [73], after listwise deletion of variables with missing information, the final sample used in this analysis consisted of 10,819 respondents of varying ethnicities residing in western China. There were 2962 ethnic minority respondents from 28 ethnic groups, namely, Tibetan, Hui, Uygur, Zhuang, Tujia, Mongolian, Dong, Yi, Miao, Buyi, Kazak, Yao, Man, Dai, Tu, Hani, Bai, Dongxiang, Alkiz, Mulao, Kelao, Li, Lahu, Shui, Naxi, Daur, Qiang, and Jing.

### Variables

#### Dependent measure

The dependent variable, self-rated health, was measured using the responses provided in the questionnaire. Respondents were asked to answer the following question: “Would you say your health is ‘very good’, ‘good’, ‘fair’, ‘poor’ or ‘very poor’?”. Responses were classified into two categories: ‘poor health’ (poor, very poor) and ‘good health’ (fair, good, very good) [74, 75].

Although self-rated health is a subjective measure of health status, it is a valid predictor with good reliability [76, 77]. Compared with many other objective measures of health, self-rated health is a robust predictor of mortality and morbidity [74, 75]. It has been widely used in studies of health research [21, 44, 73–75].

#### Independent measures

According to the study aims and hypotheses, independent measures were classified into two groups: independent variables for the primary study aim and independent variables for the secondary study aims.

#### Independent variables for the primary study aim

To the best of our knowledge, human geographic studies have seldom related ethnicity to health inequality in China. In view of this, ethnicity (Minzu) was the main explanatory variable, which was self-reported by respondents with 56 ethnic identities that have been defined in China. These ethnic divisions remained unchanged throughout the study period. Due to the small ethnic minority subsample sizes, we collapsed responses regarding ethnicity into two ethnic categories: Han Chinese and ethnic minority (the other 28 ethnicities). We recoded this measure into two categories, where 1 = Han Chinese and 0 = Ethnic minority. It should be noted that we collapsed the other 28 ethnic minorities into one category; thus, the results of this study are applicable to ethnic minorities as a whole rather than to any specific ethnicity (Minzu).

#### Independent variables for the secondary study aims

##### 1. Socio-demographic characteristics

Three measures were selected to examine the association between demographic characteristics and health: age, gender, and marital status. Age was included and categorized as “young”, “middle aged” and “older”. Marital status was categorized as “partnered” (married or de facto) and “non-partnered” (single, widowed, divorced or separated).

##### 2. SES measures

SES was mainly measured by two indicators: educational level and subjective social status (SSS). SSS refers to an individual’s subjective perception of his/her social status in society. It has been widely used in health research, and a positive association exists between subjective social status (SSS) and self-rated health [21, 78, 79]. Moreover, research has already concluded that this association is not caused by common method bias [78]. SSS was measured by asking respondents, “In our society, the socioeconomic statuses of some groups are at the top, and those of some groups are at the bottom. Which level do you think reflects your socioeconomic status?” Respondents provided their responses on a scale from 1 to 5: “lower,” “lower-middle,” “middle,” “upper-middle,” and “upper.” Educational level was classified into 5 groups (illiterate, primary school, middle school, senior high school or secondary technical school, and university and above).

##### 3. Health behaviour measures

Health behaviour variables were measured based on respondents’ self-reported alcohol consumption, current smoking status, and physical activity. It has been widely recognized that health behaviour influences health. Non-smoking, lower-risk alcohol consumption and more physical activity have been associated with lower levels of psychological distress and improved self-rated health [80–82]. Alcohol consumption was measured based on the frequency of drinking behaviour. Respondents were classified as non-drinkers, low-risk drinkers, moderate-risk drinkers, and high-risk drinkers. Current smoking status was measured with a question asking respondents about the number of cigarettes smoked every day, and each respondent was classified as a “current smoker” or “non-smoker”. Physical activity was measured by the following question: “In the last half year, did you participate in any physical activity, such as playing ball, doing Tai Chi, or practising Chinese martial arts?” Responses were categorized as regularly/every day, several times a week, several times a month, once a month or less, and never.

##### 4. Health problem measures

The impact of chronic diseases on overall population health is growing. According to the WHO global report, 80% of chronic disease deaths occur in low- and middle-income countries, where most of the world’s population lives [83]. Respondents reported their current physical condition from the chronic disease list in the questionnaire. Respondents were classified into 2 categories: person with a chronic disease and non-sufferer.

### Statistical analysis

Statistical analysis was performed by using the SPSS version 20 software for Windows, with 95% confidence intervals. Descriptive statistics were calculated, and variables related to sociodemographic aspects, socioeconomic status and health aspects (age, gender, marital status, education, subjective social status, smoking, alcohol consumption, physical exercise and chronic disease) were treated as categorical variables. All categorical variables were expressed as percentages. First, we used binary logistic regression to explore the associations between ethnicity and self-rated health. Second, for statistically significant associations between ethnicity and self-rated health, we then examined possible mediation by age, gender, marital status, education, subjective social status, smoking, alcohol consumption, physical exercise and chronic disease.

## Results

### Descriptive characteristics of the sample

Table 2 presents the descriptive characteristics of our sample. The final sample consisted of more Han Chinese (73%; *n* = 7857) than ethnic minorities (27%, *n* = 2962), and the mean age of the respondents was 45.1 years (SD = 14.8 years; range 18–99 years); 14% (*n* = 1520) of respondents reported their health as “poor”. Regarding self-rated health, more ethnic minorities (15.7%) than Han Chinese (13.4%) rated their health status as poor. The majority of respondents were married or had partners. Only 9.9% had a college/university degree. Ethnic minorities were almost twice as likely as Han Chinese to be illiterate. Only 16.7% of ethnic minorities had received a senior high school education or above, and less than half of Han Chinese had completed senior high school. The number of ethnic minorities who had a college/university degree was only half that of Han Chinese. More than half (56.4%) of the respondents reported a lower-middle or lower social status. A total of 21.9% of ethnic minorities and 31.4% of Han Chinese ranked themselves as being at the bottom of society, while 51.1% ethnic minorities and 40.8% of Han Chinese ranked themselves at the middle level of society or above. Overall, ethnic minorities rated their social status higher than did the Han Chinese. Nearly half (50.50%) of respondents never participated in any physical exercise, such as playing ball, doing Tai Chi, or practising Chinese martial arts. A total of 62.7% of ethnic minorities and 45.8% of Han Chinese never participated in any physical exercise. More women (20.3%) than men (12.4%) were illiterate. Regarding healthy behaviour, only 4% of women currently smoked, while 63.5% of men smoked. Additionally, 14.9% of men reported high-risk alcohol consumption, which was 10 times the percentage of women who reported being high-risk drinkers (1.4%). In terms of health problems, more women (34.6%) than men (28.3%) had chronic disease.

**Table 2:** Sample Characteristics (*n* = 10,819)

		Ethnicity				Whole sample		*P*
		Ethnic minority		Han Chinese				
		No	N %.	No	N %.	No	N %.	
**Self-rated Health**	Poor health	466	15.7%	1054	13.4%	1520	14.0%	0.002
	Good health	2496	84.3%	6803	86.6%	9299	86.0%	
**Age**	Young	1421	48.0%	2934	37.3%	4355	40.3%	0.000
	Middle aged	1336	45.1%	4020	51.2%	5356	49.5%	
	Old	205	6.9%	903	11.5%	1108	10.2%	
**Gender**	Female	1333	45.0%	4004	51.0%	5337	49.3%	0.000
	Male	1629	55.0%	3853	49.0%	5482	50.7%	
**Marital Status**	Non-partnered	538	18.2%	1424	18.1%	1962	18.1%	0.962
	Partnered	2424	81.8%	6433	81.9%	8857	81.9%	
**Education**	Illiterate	847	28.6%	916	11.7%	1763	16.3%	0.000
	Primary school	902	30.5%	1891	24.1%	2793	25.8%	
	Middle school	717	24.2%	2505	31.9%	3222	29.8%	
	Senior high school	324	10.9%	1646	20.9%	1970	18.2%	
	College/university or above	172	5.8%	899	11.4%	1071	9.9%	
**Subjective Social Status (SSS)**	Lower	648	21.9%	2466	31.4%	3114	28.8%	0.000
	Lower-middle	798	26.9%	2186	27.8%	2984	27.6%	
	Middle	1307	44.1%	2869	36.5%	4176	38.6%	
	Upper-middle	185	6.2%	304	3.9%	489	4.5%	
	Upper	24	.8%	32	.4%	56	0.5%	
**Smoking Status**	Current smoker	2056	69.4%	5070	64.5%	7126	65.9%	0.000
	Non-smoker	906	30.6%	2787	35.5%	3693	34.1%	
**Alcohol Consumption**	High-risk drinker	214	7.2%	679	8.6%	893	8.3%	0.000
	Moderate-risk drinker	360	12.2%	1062	13.5%	1422	13.1%	
	Low-risk drinker	420	14.2%	1535	19.5%	1955	18.1%	
	Non-drinker	1968	66.4%	4581	58.3%	6549	60.5%	
**Physical Exercise**	Regularly/every day	476	16.1%	2029	25.8%	2505	23.2%	0.000
	Several times a week	208	7.0%	891	11.3%	1099	10.2%	
	Several times a month	181	6.1%	662	8.4%	843	7.8%	
	Once a month or less	240	8.1%	673	8.6%	913	8.4%	
	Never	1857	62.7%	3602	45.8%	5459	50.5%	
**Health Problems**	Person with chronic disease	918	31.0%	2482	31.6%	3400	31.4%	0.551
	Non-sufferer	2044	69.0%	5375	68.4%	7419	68.6%	

### Ethnic disparities in self-rated health

In Table 3, we show the percentage of respondents reporting poor or good health by ethnicity. A comparison of the sociodemographic characteristics of ethnic minorities and Han Chinese revealed that over one-third (39.5%) of elderly ethnic minorities reported their health to be poor, and 7.9% of young ethnic minorities reported poor health. Meanwhile, 29.6% of elderly Han Chinese reported their health to be poor, while only 3.9% of young Han Chinese reported poor health. There were obvious statistically significant ethnic disparities between Han Chinese and ethnic minorities among the young and the elderly. Han Chinese men were the least likely to report poor health (12.1%), while ethnic minority women were the most likely to report poor health (18.8%). Regardless of ethnic differences, in general, women were more likely than men to rate their health status as poor. A total of 18.4% of non-partnered ethnic minorities reported poor health, which was slightly higher than the percentage of partnered ethnic minorities who reported poor health (15.1%). However, such differences in marital status did not exist among Han Chinese.

**Table 3:** Self-rated Health Status by Ethnicity (Minzu)

	Ethnic minority			Han Chinese		
	Poor health	Good health	*P*	Poor health	Good health	*P*
Age			.000			.000
Young	7.9%	92.1%		3.9%	96.1%	
Middle aged	20.4%	79.6%		16.8%	83.2%	
Old	39.5%	60.5%		29.6%	70.4%	
Gender			.000			.001
Women	18.8%	81.2%		14.7%	85.3%	
Men	13.3%	86.7%		12.1%	87.9%	
Marital Status			.060			.865
Non-partnered	18.4%	81.6%		13.6%	86.4%	
Partnered	15.1%	84.9%		13.4%	86.6%	
Education			.000			.000
Illiterate	25.5%	74.5%		32.1%	67.9%	
Primary school	17.3%	82.7%		19.6%	80.4%	
Middle school	7.3%	92.7%		10.5%	89.5%	
Senior high school	9.9%	90.1%		6.1%	93.9%	
College/university or above	5.8%	94.2%		2.9%	97.1%	
SSS			.000			.000
Lower	25.3%	74.7%		21.0%	79.0%	
Lower-middle	19.4%	80.6%		11.9%	88.1%	
Middle	10.0%	90.0%		8.4%	91.6%	
Upper-middle	8.6%	91.4%		8.2%	91.8%	
Upper	0.0%	100.0%		21.9%	78.1%	
Smoking			.001			.192
Current smoker	12.5%	87.5%		12.7%	87.3%	
Non-smoker	17.2%	82.8%		13.8%	86.2%	
Alcohol			.000			.000
High-risk drinker	9.3%	90.7%		8.2%	91.8%	
Moderate-risk drinker	9.2%	90.8%		5.8%	94.2%	
Low-risk drinker	9.5%	90.5%		9.2%	90.8%	
Non-drinker	19.0%	81.0%		17.4%	82.6%	
Physical Exercise			.000			.000
Regularly/every day	14.5%	85.5%		15.4%	84.6%	
Several times a week	6.2%	93.8%		7.7%	92.3%	
Several times a month	11.0%	89.0%		5.6%	94.4%	
Once a month or less	12.1%	87.9%		7.7%	92.3%	
Never	18.0%	82.0%		16.2%	83.8%	
Health Problems			.000			.000
Person with chronic disease	41.5%	58.5%		36.0%	64.0%	
Non-sufferer	4.2%	95.8%		3.0%	97.0%	

More illiterate Han Chinese (32.1%) reported poor health than illiterate ethnic minorities (25.5%). We observed that with the increase of years in school, the percentage of reporting poor health sharply decreased by nearly 30 and 20% for Han Chinese and ethnic minorities, respectively. Respondents who ranked their subjective social status (SSS) as middle or upper-middle class basically showed the same trend in self-rated health among either Han Chinese or ethnic minorities.

For ethnic minorities, there was not much difference in the percentage of respondents who reported good health among the high-risk (90.7%), moderate-risk (90.8%) and low -risk (90.5%) drinkers. However, for Han Chinese, 94.2% of moderate-risk drinkers reported good health, which was slightly higher than the percentages of high-risk (91.8%) and low-risk (90.8%) drinkers. Among current smokers, ethnic minorities and Han Chinese presented similar patterns of self-rated health. Ethnic minorities who had chronic disease (41.5%) were more likely to report poor health than their Han Chinese counterparts (36%).

### Logistic regression model of self-rated health

The primary aim of this study was to estimate the association between ethnic disparity and self-rated health. Therefore, we examined disparities in self-rated health independent of ethnicity (Model 1), sociodemographic factors (Model 2), socioeconomic status (Model 3), health behaviour factors (Model 4) and health problem factors (Model 5) (

**Table 4:** Self-rated Health Logistic Regression Models

	Model 1		Model 2		Model 3		Model 4		Model 5	
	ß	SE	ß	SE	ß	SE	ß	SE	ß	SE
**Ethnic minority**	−.187**	.060	−.415***	.064	−.243***	.068	−.193**	.070	−.222**	.077
**Age**										
Young			2.257***	.096	1.816***	.102	1.761***	.105	.981***	.115
Middle aged			.771***	.076	.590***	.081	.547***	.082	.238**	.090
**Women**			−.412***	.058	−.287***	.061	−.091	.080	.035	.088
**Non-partnered**			−.267**	.077	−.190*	.080	−.234**	.081	−.244**	.089
**Education**										
Illiterate					−1.768***	.184	−1.523***	.187	−1.334***	.197
Primary school					−1.344***	.181	−1.145***	.184	−1.015***	.193
Middle school					−.779***	.183	−.605**	.185	−.574**	.195
Senior high school					−.373	.196	−.260	.197	−.242	.207
**SSS**										
Lower					−.488	.425	−.513	.431	−.291	.486
Lower-middle					−.006	.426	−.061	.432	.204	.487
Middle					.489	.426	.448	.432	.572	.487
Upper-middle					.567	.456	.508	.461	.611	.517
**Current smoker**							−.099	.086	−.077	.095
**Alcohol**										
High-risk drinker							.831***	.137	.499**	.149
Moderate-risk drinker							.817***	.121	.569***	.131
Low-risk drinker							.485***	.093	.407***	.101
**Physical exercise**										
Regularly/every day							.125	.074	.214**	.081
Several times a week							.375**	.129	.373**	.140
Several times a month							.416**	.152	.405*	.164
Once a month or less							.196	.131	.149	.143
**Person with chronic disease**									−2.498***	.077
Intercept	1.865***	.033	1.117***	.075	2.287***	.460	1.809***	.472	3.266***	.527
Nagelkerke R^2^	.002		.118		.195		.210		.397	

Significance codes: *P < 0.05, **P < 0.01, ***P < 0.001. Reference categories: 1—Older; 2—Man; 3—Han Chinese; 4—Upper; 5—College/university or above; 6—Non-drinkers; 7—Never; 8—Non sufferer; 9—Partnered; 10—Non-smoker

Table 4). In general, all models presented similar findings (Table 3) of a significant difference in self-rated health between ethnic minority and Han Chinese. Our primary hypothesis that ethnic minorities’ self-rated health would be significantly worse than that of Han Chinese, who were expected to have better social economic status (SES), was statistically supported (P<0.001 in Model 3). We found that sociodemographic factors explained a large part of the difference between ethnic minority and Han Chinese self-rated health. Compared to Han Chinese, ethnic minorities were more likely to report poor health, and the likelihood increased by 22.8% between Model 1 and Model 2. The self-rated health status of young and middle-aged people was better than that of older people. Hypothesis 2 was statistically supported. The correlation between female gender and self-rated health was strong in Model 2 and Model 3. However, this correlation disappeared when health behaviour and health problem factors were introduced into Model 4 and Model 5.

Non-partnered patients were more likely to report poor health, which was consistent with hypothesis 4. Among the education groups, being illiterate and having a primary school and middle school education level consistently had significant effects in Model 3, Model 4 and Model 5, indicating that self-rated health was correlated with educational level. An increase in years in school decreased the likelihood of reporting poor health, and hypothesis 5 was statistically supported. For SSS, we observed a consistent pattern in Model 3, Model 4 and Model 5. In each model, ethnic minorities who ranked themselves as lower or lower-middle class were more likely to report poor health than those who ranked themselves as middle class or higher, but this difference was never statistically significant. That is, there was no statistically significant difference in the relationship between SSS and self-rated health by ethnicity.

The results of Model 4 still showed an ethnic difference between Han Chinese and ethnic minorities in self-rated health. The health behaviour factors explained the difference in self-rated health between Han Chinese and ethnic minorities. However, the difference was not as large in Model 4 as it was in Model 1. For alcohol consumption, we observed a consistent pattern in Model 4 and Model 5, and there was a strong correlation between alcohol consumption and self-rated health. Among Han Chinese, those who drank were less likely to report poor health, which was opposite to hypothesis 8. For smoking, there was no statistically significant difference in the relationship between smoking and self-rated health by ethnicity. Regarding physical exercise, we observed that those who exercised were less likely to report poor health, so hypothesis 9 was statistically supported. There was a statistically significant ethnic difference in the relationship between physical exercise and self-rated health.

In terms of health problem factors, an ethnic disparity in self-rated health existed. People who had chronic disease were more likely to report poor health, which was consistent with hypothesis 10.

## Discussion

The primary aim of this study was to estimate the association between ethnic disparity and self-rated health, and the secondary aim was to identify the influencing factors that might bring about health inequality in western China. Our results showed a positive association between ethnic disparities and self-rated health. Although the magnitude of this association slightly varied when more control variables were stepwise introduced into the models (Model 2 to Model 5), there were always significant ethnic difference from Model 1 to Model 5. We found that age, marital status, education level, alcohol consumption, physical exercise and chronic disease all had a consistent effect on the relationship between ethnic differences and self-rated health. The self-rated health appeared to be independent of subjective social status (SSS) and smoking behaviour; these findings were not in line with previous studies [80–83].

It is not surprising that the elderly are more likely to suffer from and report a poor health status. China currently has a rapidly ageing society, and the demographic structures and health status of ethnic minorities observed in the CSSC2010 database might be exacerbated over time. With rapid urbanization, a growing number of ethnic minorities have experienced dramatic changes in their ways of life. For some ethnic minorities, a semi-nomadic way of life has become only a memory, as they have moved into multi-storied houses in concentrated community. Such lifestyle changes raise questions, such as whether changes in dietary patterns affect ethnic minorities’ health and increase the risk of obesity or other diseases. Therefore, it is of great practical significance to conduct a follow-up survey to obtain an updated view of the disparity between ethnic minorities and Han Chinese.

In previous studies, socioeconomic status (SES) was identified as a determinant of health inequality [20] [23]. The results of the present study showed that subjective social status (SSS) had no significant effects in Models 3 to 5. Therefore, strictly speaking, SES could not be the determining factor of self-rated health in our study. A possible explanation is that different measurements of SES might affect the results; for example, income, occupation, family-owned luxury goods, or bank deposits are some variables that have often been used to measure SES [8].

The World Health Organization showed that chronic diseases such as heart disease, stroke, cancer, chronic respiratory disease and diabetes were by far the world’s leading causes of morbidity and mortality [84, 85]. The incidence of chronic disease in China has been on the rise, and there are obvious differences between urban and rural areas. Diseases such as high blood pressure, coronary heart disease, and diabetes and disc disease are higher among urban residents, while gastroenteritis and other motor system diseases are more prevalent among rural residents [19] [86]. Ethnic minorities often inhabit economically underdeveloped and geographically remote areas, and medical and health resources are relatively scarce. A lack of chronic disease prevention knowledge could also impact ethnic minorities’ health status. In addition, ethnic differences have been observed in cognition of illness or disease. A survey of ethnic minorities in Guizhou Province in southwestern China showed that only 38.5% of people fully agreed that regular health checks were necessary and good for health. If they became sick, many of them would not stop working or ask for treatment until they could no longer get out of bed [87].

Cultural traditions can impact individuals’ health behaviour. For Han Chinese, the percentage of moderate-risk drinkers was higher than that of high-risk drinkers and low-risk drinkers, which might be due to the unique thinking related to the Doctrine of the Mean (Zhongyong) of Confucianism in Chinese culture. The Doctrine of the Mean emphasizes moderation and suggests that going beyond is as wrong as falling short. It is believed that drinking a little every day is good for health. The adage “no gift but wine” embodies the hospitality of ethnic minorities. Wine is a necessity on important occasions, such as religious ceremonies, wedding or burial ceremonies, and for hosting guests.

This study was designed to establish a baseline association between ethnic disparities and self-rated health and its influencing factors. Future research should further explore the process through which ethnic minorities are more likely to experience poor health outcomes than non-minorities. Future research should also focus more on the spatial distribution characteristics of ethnic minorities and their impact on ethnic minority health outcomes. In addition, future research should focus on the cultural uniqueness and acculturation of ethnic minorities related to perceptions of illness, subjective social status, and health status. Of course, one limitation of our research is that the imbalance in the independent variables for the secondary study aims between Han Chinese and ethnic minorities, except for the variables of marital status and health problems, may have led to bias in the estimation of causal effects. Another limitation is that the data used in this study were collected in 2010, so we acknowledge that our findings are not perfectly valid in the current context. Nonetheless, we attempted a first step towards the identification of the existing state of racial disparities in health outcomes, which is a step that we hope will be followed by other studies in the future.

## Conclusion

This study demonstrated that ethnic disparities in self-rated health did exist in western China and that ethnic minorities who were elderly, were non-partnered, had an educational level lower than middle school, and had chronic disease had higher odds of poor self-rated health. A novel finding was that self-rated health appeared to be independent of subjective social status (SSS) and smoking behaviour; these findings were not in line with previous studies. There was no statistically significant ethnic difference in the relationship between SSS and self-rated health or for the relationship between smoking behaviour and self-rated health. Whatever the reasons might be, the inconsistent findings suggest that additional future studies are needed to explore the potential ethnic differences in the relationship of self-rated health with SSS and with smoking behaviour. Few studies have examined ethnic disparities in self-rated health in western China, and this study added to the extant research by providing an essential understanding of ethnic minorities’ health status and influencing factors. An accurate understanding of ethnic minorities’ health status in western China should not be overlooked in the achievement of the Health China 2030 plan and the 2030 sustainable development goals.

## Data Availability

The data that support the findings of this study are available from Sociology IESSR, Xi’an Jiao tong University, but restrictions apply on the availability of these data, which were used under license for the current study and are thus not publicly available. However, the data are available from the authors upon reasonable request and with the permission of Sociology IESSR, Xi’an Jiao tong University.

## Abbreviations

CSSC: China Survey of Social Change
SSS: Subjective Social Status
SES: Socioeconomic Status
PRC: People’s Republic of China
WHO: World Health Organization
